# ‘Guidance should have been there 15 years ago’ research stakeholders’ perspectives on ancillary care in the global south: a case study of Malawi

**DOI:** 10.1186/s12910-023-00889-x

**Published:** 2023-02-10

**Authors:** Blessings M. Kapumba, Deborah Nyirenda, Nicola Desmond, Janet Seeley

**Affiliations:** 1 London School of Hygiene and Tropical Medicine, London, UK; 2 Malawi-Liverpool Wellcome Trust Clinical Research Programme, P.O. Box 30096, Chichiri, Blantyre, Malawi; 3 Liverpool School of Tropical Medicine, Liverpool, UK

**Keywords:** Ancillary care, Ethics, Obligation, Consideration, Resource-constrained settings, Southern Africa

## Abstract

**Background:**

Medical researchers in resource-constrained settings must make difficult moral decisions about the provision of ancillary care to participants where additional healthcare needs fall outside the scope of the research and are not provided for by the local healthcare system. We examined research stakeholder perceptions and experiences of ancillary care in biomedical research projects in Malawi.

**Methods:**

We conducted 45 qualitative in-depth interviews with key research stakeholders: researchers, health officials, research ethics committee members, research participants and grants officers from international research funding organisations. Thematic analysis was used to analyse and interpret the findings.

**Findings:**

All stakeholders perceived the provision of ancillary care to have potential health benefits to study participants in biomedical research. However, they also had concerns, particularly related to the absence of guidance to support it. Some suggested that consideration for ancillary care provision could be possible on a case-by-case basis but that most of the support from research projects should be directed towards strengthening the public health system, emphasising public good above individual or personal benefits. Some researchers and ethics committee members raised concerns about potential tensions in terms of funding, for example balancing study demands with addressing participants’ additional health needs.

**Conclusion:**

Our findings highlight the complexities and gaps in the guidance around the provision of ancillary care in Malawi and other resource-constrained settings more generally. To promote the provision of ancillary care, we recommend that national and international guidelines for research ethics include specific recommendations for resource-constrained settings and specific types of research.

**Supplementary Information:**

The online version contains supplementary material available at 10.1186/s12910-023-00889-x.

## Introduction

Conducting research in settings where participants have complex health, social and economic needs leaves researchers with difficult moral decisions about how to respond to the needs, which may be outside the scope of the research project [1]. The ancillary care (AC) provided to participants in medical research is defined as: ‘care which is not required to make a study scientifically valid, to ensure a trial safety, or to redress research injuries.’ [2] or ‘care not required by sound science, safe trial conduct, morally optional promises, or redressing subject injury’ [3]. In 2012, Richardson redefined ancillary care as ‘medical care that the research subjects need, but that is not required to make a study scientifically valid, to ensure a study’s safety, or to redress research injuries’ [4 p.2–3]. According to Richardson [4], the definition of ancillary care clarifies that the purpose of providing this care, which is beyond the scope of the research or otherwise unrelated to the condition being studied, is to promote the health and well-being of study participants, as emphasised in research ethics guidelines [5–7]. This care could be in different forms, including direct care provision to the participants and support with diagnostic and/or other clinical services. While a growing literature on ancillary care has primarily focused on the ethics of researchers providing that care, little is known about the actual practice of AC in research settings in resource-constrained settings (RCS). Whether such care is available has implications for research participants, the health system, the conduct of the research and the regulatory and policy framework.

There are concerns that the local healthcare system in many RCS is unable to meet the healthcare needs of the population [8, 9]. Populations in these settings may be affected by poverty [10], lack of health insurance [11–13], and a disproportionate share of health conditions such as HIV, tuberculosis, malaria and the recent rise in non-communicable diseases [14, 15]. Therefore, medical researchers conducting their research in these settings may encounter unmet health needs among their research participants that may require medical care unrelated to the study. Such situations pose difficult ethical questions about the ethical principles which underpin the provision of AC during medical research and the nature of the moral implications of researchers providing AC to study participants. Olson [8] has argued that if medical researchers in RCS do not provide AC themselves or facilitate its provision by others, the health needs of their research participants may not be met, and their well-being may be compromised. The Council for International Organisations of Medical Sciences [7] includes in chapter 4 of their guidelines the statement that: “researchers have an ethical obligation to care for participants’ health needs during research and, if necessary, for the transition of participants to care when the research is concluded” [7 p.44]. The challenge, however, is that while this recommendation is given, it is not always clear what it means in practice and the scale of such care.

In a review of the practices of AC and a discourse analysis of how the language of AC has changed over time in guidance documents [16, 17], we have shown that existing guidance for the provision of AC is unclear or unavailable in the majority of RCS. We also found that researchers who take the initiative to provide AC to their study participants do so on an individualised basis or on humanitarian grounds. In the absence of defined ethical guidelines, the complexity of the obligation of researchers to provide AC remains undervalued. A review of publicly available institutional guidance documents that are pertinent to AC showed that of the 23 institutions that explicitly took a position on AC, 21 advised researchers and partners to take some measures to consider AC, and 14 specifically recommended referral for AC to local health care services [18]. The provision of AC during medical research may give health benefits to study participants [19, 20], but the practical implications of providing that care in RCS will differ from place to place

Despite the call for AC considerations in medical research gaining prominence, it is evident that guidance on its practicality in RCS remains inexplicit [17]. In 2016, Merritt and colleagues [21] proposed a framework for AC referral planning. Their framework provides guidance on deliberations researchers could use when making decisions to refer their participants for AC during medical research in RCS. Merritt suggested two ethical questions that users (researchers) can ask regarding referral for AC: (1) what impact would AC have on the well-being of those (participants) that are being referred for care, and (2) what is the impact that AC may have on local people outside the research. Merritt and colleagues were concerned by the potential challenges that researchers and members of study ethics committees may have when trying to determine what criteria constitute an appropriate referral for AC.

In the current study, we look beyond referral planning to the provision of direct or diagnostic care to study participants, as has been recognised in the theoretical literature on the topic [2, 4, 19]. In addition, we aimed to explore the perspectives of research stakeholders on the potential impact AC would have on the health of research participants, non-participants, the research and health system, and policy and regulatory frameworks. We have selected one RCS in Sub-Saharan Africa to explore differing perspectives on AC within a national setting. Malawi is a Lower- and Middle-Income Country ranked 174 out of 189 countries on the Human Development Index. Malawi’s population is disproportionately affected by severe and persistent poverty, with 52.3% reporting inadequate access to health care, according to the 2020 Integrated Household Survey [22]. The country also faces significant health challenges due to the health system’s limitations. Health care services are provided by the public and private sectors, with the government providing free services at the point of access in public health facilities. However, the essential health package, which may appear to exist only on paper, does not correspond to actual practice. In general, access to basic health care services, including screening or diagnostics and treatment, is limited. The country is heavily reliant on development aid, which plays a significant role in the economy and accounts for more than 60% of overall support in the health sector, including in health research [23].

The findings we report here are drawn from research stakeholders involved in or funding biomedical research in Malawi who provided their perspectives on the provision of AC. This paper contributes to a small but growing literature on AC in RCS.

## Methods

### Study design

We used qualitative in-depth interviews with a purposively selected sample of research stakeholders to examine experiences of and perspectives on AC provision in medical research conducted in Malawi. We examine the perspectives of a wide range of key stakeholders with diverse research experiences on the practical implications of AC. In this study, we considered all the different forms of AC offered to the individuals who participated in clinical and community-based studies. This would include the provision of treatment and/or support with other ancillary health care services such as diagnostic or referral services.

### The study setting and population

#### Context

This study was conducted at the Malawi-Liverpool Wellcome Trust Clinical Research Programme (MLW), Southern Region, Malawi. The MLW is affiliated with Kamuzu University of Health Sciences (KUHeS). Researchers from MLW conduct research and intervention studies in various rural and urban districts such as Blantyre (primarily urban), Chikwawa (primarily rural), Zomba (semi-urban), and Mangochi (primarily rural) in Malawi. The researchers are engaged in a wide range of interdisciplinary research, covering clinical, basic science, epidemiological, and public health aspects of Malawi’s most prevalent paediatric and adult diseases.

#### Ethics review and approval process in Malawi

All studies conducted at MLW and other research institutions in Malawi are reviewed and approved by the independent local (College of Medicine Research Ethics Committee and/or the National Health Service Research Ethics Committee) and international scientific and ethical review committees, depending on affiliation or sponsoring institution. These ethics committees are tasked with protecting the rights and well-being of research participants by ensuring that ethical concerns in research are minimised and that researchers rigorously adhere to ethical principles and frameworks [24]. Both local research ethics committees (REC) derive their authority from the National Commission of Science and Technology, which oversees the development of local research ethics guidelines and regulates the conduct of research in Malawi [25]. Since the MLW research is embedded within the local health system across tertiary, district and primary health facilities and communities, permission is also sought from the District Health Office or the Queen Elizabeth Central Hospital (QECH) research committees.

#### Sample

The key stakeholders in this study included researchers, health officials (both at the ministry and district level), REC members, research funding organisation officials, and research participants from purposively selected research projects at MLW (see Table 1). The selection of participants who took part in this research was based on ensuring that the perspectives on ancillary care held by a diverse range of research stakeholder groups were sufficiently represented. We also ensured a gender balance among the stakeholders, as people’s viewpoints and experiences can vary based on gender. However, gender was not found to influence the findings of our study.

**Table 1:** Number of study participants by role

Participants	Function	Number of participants	
		Male	Female
REC members	Provided experience with review and approval of research studies Experience with monitoring the conduct of research	5	0
Research regulatory authority	Provided experience in the promotion and regulation of the ethical conduct of research	1	0
Principal investigators	Provided experience in the conduct of research	2	3
Frontline research staff Fieldworkers Nurses Clinicians	Provided experience in implementation research activities—working directly with research participants	2 1 2	2 4 1
Health officials District Health Office Ministry of Health	Provided insights into the management of health facilities at the district level and provision of permission for research implementation at the district level Provided overall research policy regulation	2 1	3 0
Study participants	Volunteers in medical research	6	9
Funding Partners	Provided experience on funding for research at MLW and other RCS	1	0
Total number of participants interviewed		45 (21 m & 24 f)	

Since international funding organisations fund many projects in Malawi and RCS in general, we also selected stakeholders from these organisations to gain an understanding of their perspectives on AC. Officials from these organisations were approached for an interview. Responses to the enquiry were not received from four organisations, while officials from two others indicated that they do not have any information on their approach to ancillary care, so declined the interview.

The sampling framework was designed to gain insights from diverse stakeholders with different functions. Principal investigators, frontline research staff, and study participants were selected from ongoing research studies at the time the interviews for this study were conducted. When selecting the research studies to use as case studies, we carefully reviewed the protocols and selected only those with *ad-hoc* AC arrangements to provide such care or support to the study participants. The research ethics guidelines and regulatory policies in Malawi (a case in most of the RCS) do not provide researchers with any guidance on providing AC to study participants during medical research [17]. There are provisions for study compensation in research ethics guidelines [26], but these should not be regarded as AS described by Richardson [4 p.115]. Accordingly, compensation for study participants was not part of our inclusion criteria for the selection of case studies. We also excluded studies that did not have plans or *ad-hoc* arrangements to provide ancillary care. We used a selection criterion to purposively select studies that would help to learn from the experiences of the research stakeholders involved in those studies and to understand their opinions towards AC provision in medical research. In addition, we considered clinical/hospital-based or community-based research, adult or child participants, and the participant’s health status (healthy versus unhealthy/sick). Using this criterion, we determined the extent to which these various elements influenced the level of AC need among participants, as well as the extent to which this informed or influenced the diverse opinions of researchers. We included: (1) clinical trials, (2) cross-sectional studies, and (3) community-based cohort studies (see Table 2).

**Table 2:** Selected case studies at MLW

Case study number & setting	Study type and the main aim	Study population	Study activities	Ad-hoc AC arrangements and provision
Case study 1 Blantyre—QECH MLW	Challenge study (clinical trial) Feasibility of empirical pneumococcal carriage in Malawi and feasibility of all measures required to determine vaccine response	Healthy adult volunteers within Blantyre (all participants interviewed in our study were healthy volunteers working at QECH)	Inoculation with pneumococcal bacteria Three days observation at an arranged accommodation Follow-up on study participants	Referral for HIV if a participant is diagnosed at recruitment Provision of care for any medical condition, even those unrelated to the study (participants are advised to report at Mwaiwathu—a private tertiary hospital). Not specific to the extent to which the study can provide support for chronic conditions
Case study 2 Blantyre—QECH	Randomised, factorial, open-label clinical trial To compare the impact on 15-day and one-year mortality of combined systematic empirical treatment against TB and Cytomegalovirus plus standard of care versus standard of care in HIV-infected infants with severe pneumonia	Children less than 12 months old living with HIV (We interviewed mothers with children recruited in this study and were all coming from within Blantyre—participants were identified during follow-up visits)	Treatment and follow-up	Trial physician support with the review of participants when admitted to the ward [direct care] Provide assisted referral Provision of other social support such as food for the participant (infant milk) and clothes (diapers) [this was considered as AC by researchers, and they thought it is an important part of care] Provide TB screening to mothers, and samples are tested at KUHeS/MLW laboratory
Case study 3 Blantyre—QECH	Cross-sectional study To investigate the impact of HIV infection on the frequency and function of Mtb-specific Polycytotoxic T cells (P-CTLs) in the lung and peripheral blood in humans	Healthy, HIV-uninfected adults Asymptomatic adults living with HIV TB patients both living with and not living with HIV	Bronchoscopy sample collection and follow-up	Provision of direct care for other conditions unrelated to the study Provide assisted referral for conditions that require a specialist opinion For example, the study team had a participant with severe anaemia, and they facilitated her admission and transfusion
Case study 4 Chikwawa—community	A prospective serological community cohort study To understand the acquisition of immunity to Non-typhoidal salmonella (NTS) and epidemiology of enteric NTS and how this immunity varies with risk factors (malaria, anaemia, malnutrition, and sickle cell disease) and geographical setting	Under-five children (we interviewed mothers who had their children recruited in the study, and they were all from Chikwawa district)	Sample collection and follow-up on participants	Support with the provision of diagnostic and treatment for non-study related conditions such as anaemia and malaria Support with the referral of children diagnosed with sickle cell disease and malnutrition to QECH and Chikwawa district hospital, respectively
Case study 5 Blantyre—health centres	Phase 3 Clinical trial To assess the efficacy in the prevention of severe rotavirus Gastroenteritis of the NRRV vaccine in comparison to Rotarix To evaluate the safety of the Non-Replicating Rota Vaccine (NRRV) vaccine in healthy infants and compare it with that of Rotarix	Healthy infants, ≥ 6 weeks and < 8 weeks of age at the time of 1st study vaccination. (We interviewed mothers who had their children recruited in the study, they were all from Blantyre and identified during recruitment day at either Zingwangwa or Limbe health centre)	Vaccination at visits 1- 4 and a blood draw at the 4^th^ visit Active surveillance of Gastroenteritis throughout the study through weekly contacts	Support participants with referrals for critical conditions Provide assisted referral in cases where a participant requires to meet a specialist [when they are also sick from other diseases] after a study follow-up visit Provide care (medical care and treatment) to participants, their mother, father, and other siblings when they are sick Provide food to participants at their scheduled visits

### Data collection procedures

Interviews were conducted between September 2021—June 2022. Interviews used a semi-structured topic guide (see Additional file 1) with open-ended questions about the views and experiences of AC. Given the unique characteristics of each stakeholder group included in this study, the topic guide was used flexibly while ensuring that relevant information was gathered. We used an iterative approach [27] throughout the data collection and analysis process, in which initial interview sessions influenced the inputs for subsequent interviews. This open discussion format during the interviews allowed us to build on our understanding from previous interviews. Participants were first asked a broad question on their thoughts about AC in medical research, followed by specific questions on what they perceived to be key ethical issues and implications associated with AC provision. We used vignettes (see additional file 1) to present to participants scenarios as examples of situations where AC may be needed to elicit their views and experiences based on that understanding [28]. A clear description of AC and some examples were given at the start, and more specific examples were provided during the interview if further clarification on the topic was required. We used vignettes with study participants because we were cognizant of participants’ potential misconceptions regarding the distinction between research and medical care [29].

All interviews were conducted by the first author (BK), a social scientist, and the interviews lasted from 25 to 60 min and were audio recorded. Written informed consent was obtained from all the participants. Where interviews were conducted virtually, either on zoom, WhatsApp call or telephone call, participants granted verbal consent for participation and digital recording of the interview. For the stakeholders who were participating in research studies (as indicated in Tables 1 and 2), we asked permission from the study principal investigators to request their participant’s involvement and then approached the participant during their study visit days. However, we arranged to meet the participants for the interviews on different days from the routine follow-up visit to their main study to try and minimise the possible influence of the researchers involved in the study they were participating in. During this visit, we explained the aim of our study and gave those that were able to read a participant information sheet. For participants who were not able to read the participant information sheet, we explained to them the details of the study and encouraged them to ask questions that they did not understand. Consent was sought on the day of the interview. We did not provide any incentives to the study participants for their participation in the interview; however, participants were reimbursed for transportation.

When subsequent interviews with each research stakeholders group introduced did not yield new insights, we concluded that data saturation had been reached and ended data collection [30, 31]. In addition to that, due to the design of the study and the roles of some stakeholders, we were not required to recruit more than two participants. For instance, there was just one individual working in the regulatory authority for research in Malawi, and we chose them based on their position and level of expertise regarding research ethics in Malawi.

### Data analysis

All audio-recorded interviews were transcribed verbatim by the first author (BK), and those conducted in Chichewa were translated into English. The analysis was ongoing during fieldwork, using an iterative approach [32] to identify emerging themes that could be clarified or explored through later data collection. We conducted thematic coding, managed using NVivo [33], using broadly defined themes (such as consideration for AC or ethical concerns for AC) and inductively derived sub-themes (such as AC levels of obligation).

BK conducted the initial open coding and later worked together with JS and ND at the time of writing. The study team met regularly to reflect on and discuss emerging themes throughout the analysis process. We used a framework analysis approach to compare the perspectives of different stakeholders by theme. In this paper, we use a descriptive narrative approach to explore relationships and patterns in the views expressed by research stakeholders and to synthesise ideas that contributed most significantly to the ethics of AC practices in Malawi.

### Ethical approval

The study was performed in accordance with the relevant guidelines and regulations. Ethical approval was obtained from the Malawi College of Medicine Research Ethics Committee—CoMREC Ethics (Ref: P.01/21/3242); and the London School of Hygiene and Tropical Medicine—LSHTM Ethics (Ref: 22890). Institutional permissions were sought from all participating institutions, and their letters of support were submitted to the CoMREC as part of the submission for study ethics review. We also sought permission from the principal investigators at MLW and the KUHeS, prior to the interviews, to speak to study participants in their respective selected studies. Additionally, the research governance approval was obtained from the MLW Clinical Research Support Unit. Informed consent was obtained from all participants who participated in this study.

## Results

The data are grouped into four broad themes, all related to the impact of providing AC: (1) on the well-being of study participants; (2) on research and the health system; (3) to study participants on the individuals outside the research; and (4) on policy and regulatory frameworks. We used these themes to order the presentation of our findings, moving from the impact on study participants to the impact on policy and the research ethics guidance framing for future AC consideration.

### The impact of providing ancillary care on the well-being of study participants

Stakeholders described three main ways in which the provision of AC would have an impact on individuals who are directly involved in medical research: the expected direct health care benefit to study participants, improvement in the referral of individuals with AC needs, increase in the risk for structural coercion [34] and undue inducement.

#### Perceived direct health care benefit

All stakeholders felt strongly that AC would offer a potential personal benefit to access health care which may not be available or limited in the public health system. A commonly raised expectation was that of participants thinking that they would get the best medical care once they joined the study. For the frontline research staff and the study participants, they thought this was also associated with the possibility of gaining quick access to medical care.

‘But there are some who always say, I want my child to be helped, I do not want to come to the hospital and stand on the line [queue] for a long time. Because on the long line [queue] there, my child can be getting sicker. But here, I just come straight, and the clinician checks on my child. So, I will prefer my child to be given health services by the researchers.’ (Frontline research staff - fieldworker).

In addition to medical care benefits that most of the participants perceived as better than that which they get from public/government hospitals or through standard routine care, some study participants asked if researchers could provide them with other support, such as food.

Most study participants interviewed were mothers whose children were the main participants. The mothers emphasised that for them, apart from the care their children receive in addition to research activities, they would like social support services such as receiving food and money rather than only medical support. In terms of medical care, they indicated that they were satisfied with the care their children received as participants but while maintaining the emphasis that the provision of social support would have more impact than just medical care:

‘…when we join the research study, we have hope that the researchers will help us. So, they are still supposed to provide treatment for any disease which they have found in the child. Because whenever we join a research study, we believe it to be as our hospital; so even if our child suffers from any kind of disease, we are supposed to go there because the doctor who is doing research on the child is the one who knows the kind of disease which the child is suffering from.’ (Study participant)

Regarding the public health benefits associated with AC, the perspectives of participants in clinical studies and those in community-based studies were similar. However, there were variations over the expected health care and support needs. In comparison to mothers who enrolled their children in community-based studies, women who enrolled their children in clinical or hospital-based studies had higher expectations regarding the level of health care and social support the researchers would provide for their sick children. Adult participants had similar views on the ancillary care expectation from the researchers.

Despite these expressed expectations, the researchers indicated that participants seldom ask directly for additional care or non-medical support during research. Frontline research staff mentioned that they encounter participants asking if their relatives or themselves would be accepted to be cared for by the researchers if they get sick from a condition not related to the study.

‘… we had some mothers who came to get recruited because they were told by their friends that we provide care to them. So, they asked us if we would be able to provide care to other children in the family when they are sick.’ (Frontline research staff - research Nurse)

None of the study participants we spoke to said that they had asked for AC; rather, they said that they were satisfied with the support/help they got from the researchers. When asked about the care that the researchers provide, some participants just said, `they (researchers) provide everything,’ while others said that the researchers had told them that they could seek care at any time when they or their children were sick.

While recognising that AC has direct health care benefits for those participating in research, stakeholders from the district health office presented a different view on the impact of providing AC on the people who participate in research. They stressed the role that they wanted researchers to play in supporting the health facilities where they were implementing their study.

‘From what I have noted with the majority of researchers, they are only there for the study; they do not want to be involved in other extra activities unless you tell them that if you do not want to help us, then we will chase (not allow their research to continue) you out of here. So, some do help because they have been told to do so and because they know that if not, then we will not give them an opportunity to do their study at our facilities.’ (Health official).

The health officials believed that AC could have a greater impact if it served the entire community and were therefore opposed to the notion that AC should only be provided to study participants.

#### Improvement in the referral of individuals with ancillary care needs

All stakeholders mentioned referral for AC as one benefit of participation in medical research as it is often considered a way of helping the participant to get care for incidental findings from the study [35] or when there is a positive screening ancillary health need. They emphasised that referrals might be considered if the researcher is unable to provide the participant with the necessary care or if the participant’s condition cannot be managed at the study site.

‘So, let us say my patient in the study was enrolled and was eligible, but while in the study, has developed a heart condition, right? I can’t treat a heart condition because my focus is on pneumonia, but I can direct my patients to the right clinic and have the clinic follow up on the heart condition; still, they can still be eligible to be in the study with the heart condition. And they have the people responsible for following that up while I continue following up on the care that we’re providing, for example, in this case, pneumonia.’ (Frontline research staff–research clinician).

Some frontline research staff mentioned that researchers support their participants with a referral for AC, which sometimes includes the provision of additional support, such as for transport. Usually, the researchers do that on their own initiative to show sympathy and solidarity.

‘Like most of the time, they come here when the baby is sick. So, whenever we are thinking of referring the child to [hospital xx], we call the office for the car, and we always escort them to the referral hospital so that there shouldn’t be some delays.’ (Frontline research staff–research nurse)

Similarly, some REC members held the same view that AC referral directly benefits study participants to get help for their identified additional health needs that could not be addressed by the researchers. However, while the REC members supported referral for AC, they also raised concerns similar to those of health officials about the limited availability of services at the facilities where the participants are being referred to.

‘I would think that referral […] should be adequate, especially where such services are readily available. However, in the event that maybe this is something that is unique in a way that the facility would not provide, because sometimes those services may not be available even at the facility where the participant is being told to go, then that’s where I would probably advise that the guidelines should step in, and maybe emphasise that the study should do something about it.’ (REC member).

On the other hand, stakeholders from the district health office, while appreciative of the referral initiatives for AC, were concerned that it could overburden the health system by increasing demand for the limited resources available at the facilities.

#### Increase the risk of structural coercion and undue inducement

The protection of the rights, safety, and well-being of participants is recognised in the international research ethics guidelines as the first obligation of researchers, above and beyond the advancement of science and the interests of society [5–7]. This provides researchers with a compelling argument for addressing the clinical needs of study participants who voluntarily contribute to the progress of medical knowledge. Concerns exist, however, that researchers may also encounter volunteers with ancillary healthcare needs and that the care provided for conditions related or unrelated to the study may be of a higher standard, which could be a form of structural coercion or undue inducement. The expectations of research participants that they may accrue benefits from taking part in research, as mentioned above, were felt to influence their decision to participate.

Stakeholders had mixed views on whether AC would be coercive to study participants. While many stakeholders, including researchers and some REC members, thought that since participants get fully informed about the study and willingly volunteered to participate, AC may not be regarded as coercion for study participation. On the other hand, some stakeholders emphasised that providing study participants with AC may, to some extent, constitute structural coercion, particularly in situations in which the participants believe that participating in research is the preferable alternative to gaining access to medical care. To avoid that, one principal investigator and some frontline research staff emphasised that they deliberately exclude such information from the participants’ information sheet or when they explain the details of the study to the participants.

‘Yes, ancillary care could be a bit coercive, and that’s why, I think, we actually don’t put it either in the informed consent, or we don’t put it out there when even talking to our study participants that we will provide ABCD. But during the course of the study, that’s when we just provide it.’ (Principal investigator)

In addition, some frontline research staff, REC members, and district health office stakeholders acknowledged that it is difficult to entirely rule out structural coercion while recognising that it is not possible to determine with certainty what motivates a person to engage in a study.

‘So, speaking of ancillary care, I’d say it’s tricky because it doesn’t matter how sugar-coated you may put it to make the participant not feel they are being coerced… yes, it might not sound as if they are coerced, but in one way or the other they may be influenced, because there are some like I said who just want to know what’s going on with them and knowing that there is this advantage to come back later if they develop a problem, seriously it’s something that’s tricky on their part really.’ (Frontline research staff–research nurse).

Undue inducement, which is usually used interchangeably with coercion, presents a concern that it compromises the voluntariness of participation in research, which is a requirement of informed consent. However, it should be noted that people in RCS have limited access to health care services, and every opportunity for having access to medical care that they see in medical research will encourage or motivate them to join the study. While the frontline research staff and principal investigators mentioned that in most cases, participants are influenced to join the study because of the associated benefits, in this case having access to medical care, they believed that this was not an issue because it is difficult to ascertain what motivates an individual to join a study. Some REC members had similar views that there are many other factors that could influence participants to take part in research, but there is not a particular issue with the provision of AC. For example, they mentioned that participants could be influenced by monetary compensation, access to medical care (assumed better care), and others for altruistic motives. In addition, a principal investigator and a representative of the research regulatory authority stated that most of the participants might end up enrolling in the study without making an informed choice due to many participants' expectations regarding medical research [36].

All participants from the selected studies said that they thought that AC is part of the research and that by joining the research, it means getting better health care services in general. It is possible that participants’ healthcare expectations, rather than misconceptions [37], arise from previous knowledge of the benefits to themselves or others of participating in the research. However, while acknowledging the benefits of accessing better health care, the study participants said that they could not be forced or unduly influenced by AC to join the study.

### The impact of providing ancillary care on the research and the health system

Stakeholders considered two critical consequences that the provision of AC may have on the research being carried out in RCS as well as on the health system in general. Specifically, stakeholders were concerned about the planning for AC and the possible burden that AC may have on research as well as the health system.

#### Planning for Ancillary care

Partly linked to the consideration for the provision of AC, the inclusion of plans for AC in research protocols and grant applications was reported to be missing by all the researchers. A REC member, health officials and stakeholders from funding organisations mentioned that it is not common practice.

‘So far, I haven’t seen any protocol that I can recall seeing a protocol that had that kind of embedded as part of the study.’ (REC member)

‘Researchers don’t include ancillary care plans in their applications for funding; if they do, then it is those that are meant to provide care for the study-related condition.’ (Research funding organisation official)

Nonetheless, the PIs mentioned that they make plans to care for their study participants not only for study safety reasons but even for any other additional health needs. This substantiated what we found in the protocols of the selected studies, where ad hoc arrangements were made for the provision of some AC to study participants. In addition, stakeholders also mentioned that since there is no specific guidance on AC, studies do not have a specific budget to cover the ad-hoc AC plan that they include in the protocols. They make sure to provide everything with the limited research budget, which is meant for study activities.

Even though it was expected that the researchers (principal investigators and frontline research staff) would mention that they include plans for AC in their study protocols, since some claimed that they provide AC, they said that they exclude such plans to prevent suspicions of their influencing the participants to join the study. However, when discussing the inclusion of plans for AC, all stakeholders primarily referred to the research budget. Participants thought that including a budget to cover AC was necessary, knowing that the provision of AC would require resources.

‘…, I think it’s very essential that at the planning level, researchers should actually budget for ancillary care.’ (Frontline research staff–study clinician).

‘But if the research team would like to include a budget line, to provide that care, either by supporting a nurse or ensuring that people who are referred are seen, […] or if the team can support the health of the research participants. That, we would be happy to support that assuming that the team had made a justification.’ (Research funding organisation official)

‘But for planning purposes, it should probably be included. Even in the budgeting aspects. Yes, […] this ancillary care-related work I think should be budgeted.’ (REC member).

Some frontline research staff also suggested that identifying AC needs during study preparation can help with planning. They thought researchers could use the established networks in sites where the majority of research takes place to identify and advise on ancillary needs of people in that setting, for example.

‘In terms of planning, I think it should be the researcher looking at the local situation. So, they should be versed with the local standards. At the same time, I also understand a little bit further in terms of while Malawi has got so many limitations, but still, there are other standards that stretch a little bit further in terms of care. So, they can use the health surveillance assistants and other people to tell them about the common health needs of people within their communities and use that for planning.’ (Frontline research staff–study coordinator/clinician).

Researchers and study participants were also asked about how the plans to provide AC may be communicated to those participating in the research. Most study participants said that researchers typically inform them of the care and support they would get as a result of their participation in research. However, while several participants said that researchers tend to make this information very explicit during recruitment, they could not specify whether or not this information included conditions necessitating AC. Two study participants, one in a hospital-based and the other one in a community-based study, recalled what had happened in recent studies:

‘When they came, before we joined, they first asked us do you agree to allow our child to join the study? And I agreed after seeing that my friends are joining and also because they told us that they will provide treatment to our children if they find them with malaria.’ (Study participant)

‘The researchers make it very clear about the care we will receive while participating in the study; for example, I remember one of the study nurses mentioning to me that I could come at any time I feel sick, and the study doctor will review me and give me medications.’ (Study participant)

However, the majority of participants could not differentiate between study-related care and AC or support but were very appreciative of all the care that they received while participating in the research.

Researchers had mixed views on whether to include AC statements in the participant’s information and when to tell participants about AC. While most of the stakeholders thought that including AC statements would unduly influence participants to take part in the study, others suggested that everything must be explained to the participants. They thought the decision must be made by the participants to either take part in a study or not. One frontline research staff thought that if researchers decide to include AC information in the consenting process, then that care should be equal to the standard of care provided in public health facilities.

#### Burdens on research and healthcare system

The view that AC would be a burden on local healthcare systems was emphasised by many researchers, REC members and health officials. They saw that this care would add extra responsibilities to the already constrained healthcare system. Health officials were concerned that there was already a lack of resources in most of the facilities; when researchers refer the participants for AC, it would, in the process, overburden the limited resources of the health system. Health officials who raised this issue believed that researchers could step in and assume some responsibility; they should not leave everything to the health system, lest the individuals they refer to the public health system be unable to get assistance.

However, one health official from the Ministry of Health thought AC would not create much of a burden to either the research or the healthcare system because each has a specific role to play in patient care.

‘… I believe that this participant or patient has already spent much of his or her time with this researcher. Now this researcher has identified the problem and referred this patient to maybe another level of care. To me, I don’t think it is a problem or burden on either the researchers or the health care system.’ (Health official).

On the part of the research, some researchers and REC members thought that giving the responsibility to researchers to provide direct AC would create an unnecessary burden on the research. Stakeholders were concerned that research resources are often restricted to study-related activities; therefore, using the same study resources for AC might deplete resources intended for study-related activities. Although the stakeholder from a funding organisation mentioned their flexibility to consider providing top-up funding for AC as it may be requested by researchers, this was not specific about what that would mean in practice or how much of the budget they would be willing to provide for AC.

### The impact of providing ancillary care to study participants on the general population

In this section, we focus on two critical viewpoints that came up in the interviews in relatione to potential impacts of AC on the broader population. While most stakeholders emphasised that providing AC may be one strategy for strengthening the local public health system, some expressed concerns that it may promote health inequities regarding access to health care.

#### Healthcare system capacity strengthening

Many stakeholders emphasised that researchers could consider providing AC as a form of providing support to the public health system. They thought the way to address healthcare challenges that the majority of study participants in Malawi experienced could be through supporting health facilities in the districts where they conduct their research.

The district health officials emphasised that they want to benefit as much as possible from the research because they assume that researchers benefit in the process of conducting their research [38]. However, one frontline researcher was against the idea of researchers supporting the health care system as a whole, arguing that AC should be focused only on an individual who has voluntarily decided to participate in the study and perhaps knows his/her problems.

‘Targeting the whole system would deprive the needed care to the individual at the point they needed that scarce service which has been offered to someone else.’ (Frontline research staff—research nurse).

Some research stakeholders mentioned that, since some research procedures use resources which are already scarce within the government health care system, for example, laboratory testing supplies such as reagents, they need to come in to support the system.

‘These supplies are usually out of stock, and if researchers know that part of their study would require that, then they should be able to plan for that and help supply such commodities to the hospital.’ (REC member)

Another aspect of social support emphasised by the district health officials was capacity building within the health care system. They said these are some of the important things they would like to see done by the researchers, which would also be considered AC. For example, teaching district health office personnel about research techniques and emerging medical technology undertaken by researchers throughout the implementation of their research activities.

#### Promote the potential for healthcare inequalities

In this category, stakeholders brought up some concerns about the potential inequality that AC may cause between individuals involved in research and those who are not. The concern about inequality was predicated on the idea that limited health care impacts everyone in RCS, not only those who engage in research and have difficulties gaining access to vital health care services. Therefore, if the provision is limited to those who engage in medical research, there is a risk of exacerbating healthcare access inequities [39]. The health officials and some REC members emphasised the need for researchers to focus on the public good versus the individual or personal benefits to address such inequalities.

However, some stakeholders thought that including people not involved in the research would be a burden on the researchers. They mentioned that for the researchers to provide AC to their participants, there are several factors, including the trusting relationship that is established between the researchers and the participants.

‘First one is the researcher has identified a problem in the participant; they don’t do that to people who are not participants in their study. Therefore, now there has been an established kind of relationship between the participant and the researcher. Therefore, the researcher should be sympathetic enough to address that challenge in the participant.’ (REC member)

The members of the REC, the researchers, and the health officials all shared the perspective that the general community could consistently profit from the results of the study.

### Ancillary care ethics, policy, and regulatory framing

#### Ethics guidance for ancillary care

The REC members, health officials and researchers all acknowledged that the current ethics guidance does not support or explicitly mention the provision of AC to study participants.

‘So, we don’t have, as of now, we don’t have any, you know, policy guidelines along those lines in terms of care. We only go by the fact that when people are doing research […] it should be beneficial to an individual directly or indirectly, or the community immediately or later on.’ (REC member).

Some REC members and one researcher mentioned that guidance for AC had been needed for some time.

‘Well, I think the guidance should have been there 15 years ago, but it wasn’t. That is not written anywhere. But I think, you know, maybe they could request that. As I say, I’ve always put ancillary care in my budget, where I use the 10% contingency.’ (Principal investigator).

Both the REC members that we interviewed and stakeholders from research funding organisations accepted that AC had not been discussed in anything but an ad hoc way and had not been included as a priority issue in their deliberation on policies.

‘I would say, maybe the only time that we start to interrogate or talk about issues to do with or that could maybe feed into ancillary care is when we are looking at adverse events.’ (REC member).

‘We have had debate on compensation, but that issue has never come up and say if they are providing care to the participants, then they will sway the participant to join that wouldn’t otherwise join. So, to me, it’s like if it is beneficial, it is difficult, rather just deal with the case on its merit.’ (REC member).

In response to our email, a representative from another research funding organisation that we did not interview stated that they do not have specific information on AC and instead refer researchers whose projects have been funded to the in-country regulations.

Some stakeholders, including the REC members and researchers, suggested that considerations to make some changes in the research ethics guidelines should happen now. One REC member mentioned that the landscape of international ethics guidelines has changed, and this must be reflected in the local research ethics guidelines to address issues of AC in medical research.

‘Okay, but putting my thoughts along those lines, I’m about to say that it should become pretty much like the guidelines; they should revisit that whole thing and maybe make it more kind of obligatory within certain kinds of boundaries. […] they should be able to consider the type of maybe support they can provide, you know, and what that is doing, but that component should really be part of any study of this magnitude. So, yeah, I’ll say that there’s need for that to make sure that it reflects on the guidelines.’ (REC member).

When we asked the researchers about the guidance for AC, they mentioned Good Clinical Practice training on participant protection. To most researchers, this is the ethics guideline for the conduct of research, mainly to safeguard and protect participants from research harm.

When asked about the availability of specific guidance from the ministry of health for researchers when they conduct medical research in Malawi, health officials mentioned that there are no specific guidelines or policies on ancillary care or for the conduct of medical research in general. However, they contribute to developing the ethics guidelines the REC members use, which they believe all researchers are supposed to follow when conducting their research.

#### Ancillary care obligations

Research stakeholders had mixed views on the obligation of AC that may require that researchers take full or some responsibility towards providing care to their participants during medical research.

While all research stakeholders expressed support for researchers to take responsibility for the provision of AC, some did not agree to make it an obligation.

An official from the Ministry of Health and other stakeholders, including the researchers, held the view that making the provision of AC an obligation for researchers would make the conduct of research in Malawi costly.

‘Making the provision of ancillary care obligatory will set up higher standards for research funding which will be difficult to sustain.’ (Health official).

‘Firstly, it shouldn’t be the researcher’s obligation. It shouldn’t be because we have specific things to do in research, and there are already people who provide care for other conditions. But if something has been stated in your protocol, that this is how we’ll do things, that should be implemented exactly the way you say it.’ (Frontline research staff–research nurse).

In addition to the concerns around the cost of making AC an obligation, some frontline research staff thought that this would put too much responsibility on researchers as well as increase the demand for care services from participants when they learn that researchers provide AC as may be deemed necessary.

‘[…] at the same time, it might even make most studies not feel like a good ground for them to practice or to do research because of the demand from participants and knowing that it’s an obligation.’ (Frontline research staff–study Coordinator).

Instead, stakeholders, including the REC members, researchers and the official from the research funding organisation, suggested that AC should be provided on a case-by-case basis because it is not all participants have additional health needs or incidental findings during medical research.

‘I think guidelines are clear around doing research and on uncovering or finding out that participants have sort of health needs, which I think are mostly kind of on an individual basis, so they should be supported as such.’ (Research funding organisation official)

For the stakeholders who held the view that AC should be an obligation, many thought that since it is not included in the guidelines, it was important to have a careful review of the guidelines and include AC obligations.

All the PIs and frontline research staff that we interviewed reported that they have an obligation to provide AC to their participants. Some of the reasons that they provided include the moral responsibility to help others.

‘Well, I think from the point of the [case study 2] trial, I feel like we find this a moral obligation, that you’re not just a thing to be experimental in our study. There are some participants with additional needs that we should support if you want to sort of them participate in our study.’ (Principal investigator).

‘It’s certainly an obligation depending on the obligation kind of research that you are doing. So, to me, maybe the higher the risk, the more ancillary care could need to be provided.’ (Principal investigator)*.*

The health officials, however, had different views on AC obligation. While indicating that researchers have an obligation to provide care to their participants, they suggested that it would be better if the researchers focused on the health system and not an individual.

## Research funding constraints

Research funding was perceived as a limiting factor in AC provision plans. Some researchers thought it is now time for research funding organisations to start considering including or accepting some budgetary plans (as proposed by applicants) for AC.

‘They need to really have an understanding of the challenges people face and plan to give researchers some additional funding for ancillary care needs which may be identified during the implementation of the study.’ (Frontline research staff - research nurse).

However, several stakeholders were concerned that it would be difficult to ask for extra money and that, given there is already usually a 10% contingency, that might be used for AC. One REC member commented that there really should be funding committed/allocated to supporting either AC or the health care system.

‘One of the things that I have found almost immoral, I am going to use that term, is that you have a study with a huge budget, and a huge proportion of that money goes in the form of the fees or payments to the PIs. A huge component of the money would remain, for instance, if it’s coming from outside this country, would be with those people that are from the other world or what is commonly known as the global north.’ (REC member).

A stakeholder from one of the funding organisations mentioned that while acknowledging that funding support towards AC may not be supported by a policy within the organisation, the importance of AC in RCS cannot be overlooked. They suggested that funding for AC may be considered, provided that researchers include a clear justification and that it does not take the whole research budget.

‘…we would expect that the team have comfortably budgeted for what they need and that they have provided some justification as to how they have arrived at the number for the cost of ancillary care.’ (Research funding organisation official)*.*

The same respondent went on to suggest that funders might provide additional funding if it is meant to support the health system.

‘If there’s some other mechanism that basically, the team can support the health of the research participants. […] we would be happy to support that, assuming that the team had made a justification. They just need to justify how they’re going to use that money and what it’s for and kind of the ethical considerations down to the participants.’ (Research funding organisation official)*.*

However, some stakeholders were against the idea of directing funders to accept all the AC plans in the grant’s application because they thought this might make the implementation of research very expensive and hence discourage research funders as well as researchers who fail to source funding for AC.

## Discussion

Our findings provide insights into the experiences and perspectives of research stakeholders regarding the provision of AC to study participants during medical research in Malawi. In this study, we found that, in theory, the stakeholders consider AC desirable, but when looked at more closely, questions arise about how applicable provision through referrals maybe if the health system is overwhelmed. There are also issues about costs when AC is not something provided for in research budgets. While funders may express the view that AC is something they could consider, the concern that this may make budgets prohibitively expensive, particularly in situations where research funding is scarce, does require careful consideration.

The historical rationale for research regulation was to protect study participants from study-related harm while also aiming to improve individual and public health through new discoveries [40]. Collectively, medical research has led to significant discoveries, the development of new therapies, and a remarkable improvement in health and public health [41]. However, often forgotten are the actual benefits that this has to the individuals who participate in medical research if they do not receive care for the additional health needs that they may have during the time they participate in research or beyond. Several studies have been undertaken to gauge public attitudes towards health research and the factors that influence individuals’ willingness to participate in medical research [29]. However, less focus has been given to understanding the impact of research on individual participants. In relation to the AC expectations of study participants, our findings are consistent with previous studies [42–44]. Our results suggest that therapeutic misconception, or, indeed, the expectation [37, 45] that many participants have regarding medical research, was the primary motivation for their engagement in research. In addition to expecting to be informed of the study’s findings, participants may also have expectations regarding their healthcare needs, especially at the time they enrol in the study or during the implementation of the study. Our findings suggest that the provision of AC in this regard may be associated with an expectation that study participants may have, such as the belief that they will have access to healthcare services that will address all their health needs, including services that are scarce or beyond the standard care. With such expectations, Sacristán et al. [46] argue that participation in research is often motivated by the possibility of personal benefits as well as the chance to assist others. In the context of RCS, despite the danger associated with exposure to experimental procedures and therapies, research participants continue to be driven by a choice to or a perceived belief that they will only get better care if they engage in research. This suggests that guidelines for AC provision might need to be different in these settings acknowledging the reality that, if given a chance, individuals would prefer to participate in research explicitly to receive access to better treatment. Similarly, our findings demonstrate that individual benefits of participating in medical research in RCS through AC would have a direct impact on the health of the participants, as suggested by Nass et al. [47].

In the context of AC referral, reported as the most common practice for AC in medical research [16], Merritt et al. [21] pointed out that researchers ought to consider the prospect of AC (referral) as a benefit to studying participants in the light of the associated burden and risks. However, taking such responsibilities means that researchers have to make proper plans to ensure that the provision of any form of AC does not impinge on the primary obligations for research [48]. In our findings, we have demonstrated that AC plans are not usually included in research protocols or where researchers are applying for research grants. Those that do take the initiative to provide AC to their participants do so on a case-by-case basis. Taylor et al. [49] suggest that an essential part of AC planning is when researchers anticipate the possibility of giving some AC based on their knowledge of the health state of the community from whom eligible subjects will be recruited. We had similar findings in our study; however, for most of our research stakeholders, the term “planning” was interpreted as referring only to the inclusion of a budget or some other form of financial allocation for activities that are considered to be AC. Because researchers do not have clear AC plans in their protocols, we assume that this was the reason why AC was not clearly explained to study participants [46].

Our findings also demonstrate that referral for AC may have been the most supported practice because it does not require unmanageable amounts of additional resources from researchers. However, a referral could be made on the premise that such health services are publicly supported by the facilities where participants are being referred, while this may not be the case. We found that additional support or non-medical support provided to study participants requiring referral for AC which was mentioned and also suggested by the stakeholders in this study, was similarly reported by Pratt et al. [39], where researchers in a trial took the responsibility of supporting referral for AC by providing transport.

In relation to the impact on the research and the health system, stakeholders’ views reflected principles and potential tensions regarding the resources that are used for the successful implementation of research. While researchers were concerned about having limited funds or restrictions in their budget and that AC would consume resources meant for the study, health officials, on the other hand, saw AC as a burden on the health system. Although stakeholders were supportive of the provision of AC during medical research, there were no clear reflections on the ethics of doing that. Many stakeholders’ viewpoints were based on the social aspect of moral obligations, that it is in the nature of human beings to help one another in situations of need. Evans, Evans [50] describes the situation of need (vulnerability) of participants in medical research as evident, and this is particularly the case with participants in RCS. Despite the fact that stakeholders saw AC as complex and demanding on either the research or the health system, we argue that its influence on both study participants and the general population is highly beneficial.

In relation to the impact on the general population, enforcing the capacity of the local public healthcare system/facility was one consideration strongly suggested by research stakeholders in this study. This suggestion is strongly supported in the [CIOMS (1992): cited in 52 p. 141] (Guideline 15), which recommends that consideration should be made to strengthening the healthcare facilities and ensuring that it is sustainable in the local context once the research has been completed. In addition to a range of activities for capacity building, such as specialised training for medical personnel working in health facilities, research stakeholders suggested that AC support may include helping with medical supplies, such as medical equipment and medications, as well as providing direct care to individuals who seek medical treatment at the health facility. Stakeholders’ viewpoints show that the AC responsibility of researchers and research funders should be directed toward strengthening the healthcare system so that it can benefit many. Taylor et al. [49] suggested that research sponsors should support the researcher’s commitment to contribute to the overall health and well-being of the community in RCS. Similarly, our findings demonstrate an emphasis by stakeholders for similar support, as they believed that if researchers were supported with sufficient funding or resources, they would be able to assist the challenged health system, with the benefit being for the public good as opposed to individual benefit [51, 52]. However, in terms of the provision of AC, researchers cannot be fully responsible for providing everything that health officials in the health system may demand from institutions involved in research. For example, the research stakeholders’ emphasis on good working relationships is to see that there is benefit sharing. Thus, the healthcare system will benefit from the resources brought by the researchers, which may be used by individuals who are not participating in the study, in addition to those who are the study’s primary beneficiaries (the study participants). In the healthcare system, there are many stakeholders who partner with the Ministry of Health, which impacts different healthcare systems/facility capacity strengthening. One example of such a stakeholder would be researchers or the research institutions to which the researchers are affiliated. As a tool to facilitate this relationship between patients (research participants or not), the researchers, and other players, with the goal of defending the patient’s rights to health care, researchers are an important stakeholder [53]. Clearly, stakeholders emphasised/perceived value in healthcare system/facility strengthening activities to support the fragile healthcare system. However, just as we described above on AC obligations, most of the stakeholders perceived this to be overburdening the research as well. They were also looking beyond the completion of the study to what would happen to the patients or participants who depended on the AC being provided by the researchers [54, 55].

In relation to the impact on policy and regulatory framing, our findings have demonstrated that ethics guidance for AC is lacking [18] and where it has been included is not explicit. As the debate on the ethics of AC provision during medical research continues, our findings suggest that the normative perspective of AC provision must be translated into practice, hence increasing the potential for the development of new guidelines. Advances in such areas of research ethics are facilitating a transformation in the ethics of research, which is generating new insights into the conduct of health research. Since the current ethics guidelines do not explicitly support the provision of AC, we contend that this increases the nonstandard method of providing AC to study participants, which further complicates an already complex issue. Lack of clear ethics guidance is also thought to make it difficult for researchers to decide on what to do when they identify AC needs in their participants [17]. Even though our findings indicate that researchers assume some responsibility for providing AC (see Table 2), there is widespread controversy and a lack of ethical guidance regarding what researchers can provide as AC and the boundaries of AC provision. Our findings have also showed that discussions around AC have not been prioritised by the research ethics regulatory bodies. Commonly addressed concerns include remuneration or compensation of study participants, such as how the researcher might avoid paying too much while determining an appropriate amount of compensation for study participants in order to avoid unduly influencing or coercing their participation. [6, 7].

Although our findings show some potential that research participants may be unduly influenced or coerced to participate in a study due to AC being provided, we argue that this is a very minimal concern, similar to the views presented by the REC members. However, according to Nkosi et al. [56], participants in RCS frequently perceive study resources as a chance to improve their lives, which undermines their decision to deny involvement in the study, which might be viewed as structural coercion in a sense [34]. It is clear that the provision of AC is still a new concept to several of those interviewed for this project, and more discussion is needed, both within the country and internationally, to agree on what guidance can be given and what should appear in policy documents to guide this provision.

This study is not without limitations. Firstly, the representation from research funding organisation partners was insufficient to provide an adequate reflection of how different research funding partners regard AC support for researchers conducting medical research in RCS through grants. Similarly, it could have been ideal to interview research stakeholders from other research institutions that conduct medical research in Malawi and get funding from different partners. Since we were more interested in interviewing officials from funders that fund medical research in Malawi, and more specifically at MLW, we believe that including funders from other research institutions in Malawi would have given other additional perspectives on AC provision. However, some officials from the funding organisations responded to our request for their involvement in the study through email by stating that they do not have any specific information on ancillary care. As the objectives of the study were related to experiences, opinions, and practices, it may be assumed that stakeholders who decided not to take part in the study or did not reply are likely to have perspectives that would support AC. From this viewpoint, stakeholders from other research funding organisations could have different opinions about what counts as AC. We may thus have missed important voices and perspectives. However, the responses we got from stakeholders (declined potential participating officials) from some funding organisations were considered as a finding for our study. Moreover, it is well established [17, 18] that the majority of research institutions and funding organisations do not have explicit guidance for ancillary care.

Secondly, the findings of this paper provide an in-depth exploration of AC practices and the related ethical challenges experienced by researchers undertaking medical research in Malawi, where research ethics guidelines are not currently explicit on the provision of AC [17]. Conducting a similar qualitative in-depth interview study in other socio-economic, cultural, social, and geographical contexts on practices of AC provision in medical research should be considered to complement and contrast our findings. Given that we identified ethical challenges associated with the implementation of ancillary care in medical research conducted in Malawi and that it is difficult to determine how much AC can be provided to a participant, future studies should explore whether similar ethical challenges associated with AC exist in settings with some AC guidelines or in contexts where research is partially locally funded (do not largely depend on international funding partners), as well as in settings where health services are not free to the public or with national health insurances. For instance, South Africa has projects financed by the South Africa Medical Research Council, whereas Kenya has projects funded by the Kenyan government. Indeed, as much as our findings represent RCS, they should be interpreted cautiously.

## Conclusion

This paper highlights the broader questions that researchers need to ask when considering the provision of AC to their study participants in medical research. Despite the best intentions of some researchers to provide AC, our findings demonstrate that the concept of AC is still new among many research stakeholders. Most of the responses from the stakeholders are that AC should be encouraged as a moral practice in research. However, the planning and provision of AC should not be mandatory. When considering the provision of AC in medical research, researchers should not limit themselves to protecting study participants from study-related harm or illness. Instead, they should adopt a broader care perspective that includes caring for their study participants’ additional health needs. In addition, standard criteria must be specified for RECs and researchers to use as guidelines when reviewing research proposals and determining the type and extent of AC that researchers can provide to study participants. Therefore, we recommend the development of a more explicit internationally agreed-upon ethical framework to guide decisions regarding AC that would be applicable to all stakeholders, including sponsors or research funding organisations from the global north. In the absence of internationally binding regulations on AC, this would also guide researchers in protecting the well-being and health of their participants in RCS.

## Supplementary Information

**Additional file 1.** Topic guide for KIIs with research stakeholders in Malawi.

## Data Availability

The datasets generated and analysed during the current study are not publicly available owing to privacy concerns; however, they are available from the corresponding author upon reasonable request.

## Abbreviations

AC: Ancillary care
CIOMS: Council for international organisations on medical sciences
KUHeS: Kamuzu university of health sciences
MLW: Malawi-Liverpool Wellcome Trust Clinical Research Programme
RCS: Resource-constrained settings
REC: Research Ethics Committee
NRRV: Non-replicating rota vaccine
NTS: Non-typhoidal salmonella
QECH: Queen Elizabeth Central Hospital
