# Supplementary material for: ‘Guidance should have been there 15 years ago’ research stakeholders’ perspectives on ancillary care in the global south: a case study of Malawi
Source: BMC Med Ethics. 2023 Feb 10;24:8. doi: 10.1186/s12910-023-00889-x (PMC9912595; doi:10.1186/s12910-023-00889-x)
Supplement: Supplementary file 1 — Additional file 1. Topic guide for KIIs with research stakeholders in Malawi. [file 12910_2023_889_MOESM1_ESM.pdf]

Additional file1: A topic guide for stakeholder interviews

The Social and Ethical Implications of Data-Prompted Ancillary Care in Southern Africa, Malawi

A. **<u>Research principal investigators and frontline research staff (MLW fieldworkers, nurses, clinicians)</u>**

Interview questions focused on the interviewee's familiarity with the concept of ancillary care and the description of the current practice and what ought to happen.

Introduction

1. To start, could you tell me about what you do, the study you are working on or what you have done before?
2. How long have you been working/involved in research or in your current position?

Research ethics guidance

So, just like with any other research project, I believe there are specific research ethics guidelines that you use or follow when conducting your medical research.

3. What specific research guidance do you use or follow for the conduct of your study? Or what specific ethics guidelines do you and your study team follow when implementing study activities?
    a. International
    b. Local
4. What guidance do the local research ethics committees provide on supporting the provision of ancillary care during medical research, particularly on research conducted in our resource-constrained settings?

In some ethics guidelines e.g., the CIOMS guidelines, they describe some consideration for the provision of care to study participants during research using terms such as morally praiseworthy, as necessary – but not calling it an obligation.

5. What do you think of such guidance statements to researchers conducting research in RCS?
    a. What do you think about the current local research ethics guidance?
6. How does that apply to the studies that you conduct or to you as a researcher?

What can you tell me about ancillary care provision in your study? or What are your experiences with the provision of ancillary care to study participants in your current or previous research?

7. What are some of the common ancillary care health needs identified among research participants? And how do you respond to them?

Ancillary care practices

8. What ancillary health needs do you expect or have you experienced in your participants?
9. What guidance do you provide in your study on supporting the provision of ancillary care during the conduct of your research?
10. What do you think about the provision of ancillary care to study participants in biomedical research in Malawi?

    - Probe for reasons
        - benefits
        - concerns/risks/challenges

11. In general, what are your views on access to care for non-study-related conditions for research participants in medical research?
12. During the designing phase of your studies, what ancillary care plans did you include in your study protocol or when applying for grants, if any? And how did that go with the ethics review?
13. Under what conditions are supported referrals of research participants with ancillary care needs made, and what are the outcomes? Or to what extent would ancillary care be provided?
14. If ancillary care is to be made available to study participants in Malawi or in RCS in general, what do you think would be the important things to put in place or what needs to be considered?
15. What ethical issues do you (as a researcher) or researchers encounter regarding the provision of care or support outside the study to their participants?
16. What are your views on the researcher's responsibility of planning and responding to ancillary care needs during research?

    - Probe for
        - What obligations do researchers have to participants?
        - What role do context and circumstance play in determining obligation?
        - How do ideas of fairness and reciprocity play out in the context of limited research funding and inadequate national health systems?

17. What if anything, should be provided to research participants (both as individuals and groups), and by who, after their participation, and what, if anything, should be made available to others in the host community or country during the research”
18. How could provision of ancillary care in Malawi or RCS generally likely impact on the research study objectives?

    - Probe for
        - Increased demand
        - Resources (finances and other study materials)

Suggestions for ancillary care in future studies

19. What are your thoughts on including plans for ancillary care in the study design?
20. What would be the necessary steps to follow if ancillary care is to be planned and provided to study participants in Malawi and other RCS more generally?
21. What do you think about the idea of ancillary care (care and treatment) in medical research?

Thank you for your time and participation in this study.

B. **Research ethics committee members (COMREC and NHSRC)**

Interview questions focused on the interviewee's familiarity with the concept of ancillary care and the description of the current ethical guidelines.

Introduction

1. To start, just tell me about your role. What position, involvement with (own) research?
2. How long have you been involved in the committee?

Views on ancillary care

3. How do you describe ancillary care? or What would you describe as ancillary care in biomedical research? What do you understand about ancillary care in medical research?
4. What do you think about the provision of ancillary care to study participants in biomedical research in Malawi?

   - Probe for reasons
     - benefits
     - concerns/risks challenges

5. What are your views on the researcher's responsibility of planning and providing access to care for non-study related conditions for research participants in medical research?

   - Probe for
     - What obligations do researchers have to participants?
     - What role does context and circumstance play in determining obligation?
     - How do ideas of fairness and reciprocity play out in the context of limited research funding and inadequate national health systems?

Research ethics guidance

6. Does the NCST have existing guidance available on supporting the provision of ancillary care during medical research, particularly on research conducted in our resource-constrained settings?

In the CIOMS guidelines, they describe some consideration for the provision of care to study participants during research using terms such as being morally praiseworthy, as necessary – but not calling it as an obligation until recently in the 2021 CIOMS guidelines where it is now being referred to as an obligation.

7. What do you think of such guidance statements to researchers conducting research in RCS?

8. What do you think about the idea of ancillary care (care and treatment) in medical research?

In the COMREC general guidelines 2010– adopted from NCST- we did not find any information that supports the provision of care to study participants including the study related. Such information is not included in the NHSRC general guidelines as well.

9. As a member of the research ethics committee, I understand you have been involved in reviewing several protocols. When reviewing these protocols, what ancillary care plans, if any, have you come across or do medical researchers include in their research protocols?
10. When reviewing protocols for health-related studies, how do you determine what researchers can provide to their participants during research?

    Is any consideration made during the review of the protocol for research when it is with participants who may have additional health needs besides those which are related to the study?

11. What ethical guidelines does REC provide to support researchers providing for the study participants' additional health needs in Malawi?

12. How could the provision of ancillary care in Malawi or RCS generally likely create ethical concerns in the conduct of biomedical research?

    Coercion

    Therapeutic misconception

13. Would NHSRC expect to see researchers setting out ancillary care plans in the researcher's ethics approval applications?

Suggestions for ancillary care in future studies

14. What would you suggest to funders of research on considerations for additional funding for ancillary care?

C. Grant officers from international funding institutions

Interview questions focused on the interviewee's familiarity with the concept of ancillary care and the description of the current research funding guidelines for RCS.

Introduction

1. To start, just tell me about your role. What position or involvement with research funding or grants?

Views on ancillary care

2. What type of research do you provide funding for?
3. What are your thoughts or opinions on the considerations for ancillary care?
   - What plans do you have (as funders) for ancillary care to study participants in RCS?
   - What position does your funding organisation have on supporting the provision of ancillary care
4. What specific guidelines, if any, do you provide (or do researchers follow) to researchers when applying for research grants?
   - How do they address issues of differences in study contexts? Of particular interest are studies conducted in RCS where its participants are vulnerable.
   - What funding considerations are there on participants' additional health needs besides a condition under investigation?

   We saw that some of the policies and guidelines for Wellcome funding refer to CIOMS, the Declaration of Helsinki and other international ethics guidelines on the conduct of research. These guidelines contain some statements on consideration for the provision of care to study participants – as morally praiseworthy or if necessary – however, these terms are not explicit. The 2021 CIOMS explicitly calls ancillary care an obligation of researchers.

   - How does that reflect on the funding that you provide for research in RCS where we expect that these needs will arise?
   - How does the research funding provided by your organisation reflect on the international ethics guidance which recommends that medical researchers can provide ancillary care to their participants if necessary or because is something praiseworthy?

   Collateral benefits - provision of healthcare benefits to communities during a research study

- What are your thoughts on such guidance statements?

5. How much ancillary care plans do you see in the grant's application? What do researchers include in their grant application regarding the support with the provision of ancillary care to the study participants?
   - What do you do when you find that researchers have included a budget for the ancillary health care of their participants?
   - How does Wellcome accommodate what researchers plan as the budget for other additional health care to their participants?
6. What are your views (or the stand of Wellcome) on the researcher's responsibility of planning and responding to ancillary care needs during research?
   - Probe for
     - Some research stakeholders with whom we have spoken have said that researchers gain a great deal from the information that they get from their participants. In this particular example, they are looking at the people who volunteer to participate in research in RCS, who are deemed vulnerable in a variety of aspects of their lives including on health. How do ideas of fairness and reciprocity play out in the context of limited research funding and inadequate national health systems?
     - What do you think of this obligation of researchers have to participants?
     - What role do context and circumstance play in determining obligation?
     - What happens with funding or what would happen to funding if researchers have exhausted their funds to take care of the ancillary health needs of their participants?
7. What are your thoughts on the ethical implications (on funding) of researchers supporting ancillary care provision to study participants in RCS?

Suggestions for ancillary care in future studies

8. What are your thoughts on researchers including plans for ancillary care in the study design?
   - When researchers are applying for funding from your organisation.
9. What would you say would be the necessary steps researchers should follow when deciding and considering the provision of ancillary care to study participants in Malawi and other RCS more generally?

D. **Health officials (Ministry of Health and District Health Offices)**

Interview questions focused on the interviewee's familiarity with the concept of ancillary care and the description of the current ethical guidelines.

Introduction

1. To start, just tell me about your role. What position, involvement with (own) research?

Views on ancillary care

2. What is your relationship with researchers like?
3. In what way would you need help from the researchers when they conduct their studies in your facilities?
4. Does the ministry have existing guidance available on supporting the provision of ancillary care during medical research?
5. What do you think about the idea of ancillary care (care and treatment) in medical research?
6. Is any provision made during the time of your considerations (**when giving permission for research**) for research when it is with participants who may have additional health needs besides those which are related to the study?
7. What do you think about the provision of ancillary care to study participants in biomedical research in Malawi?

    - Probe for reasons
        - benefits
        - concerns/risks/challenges

8. What are your views on access to care for non-study-related conditions for research participants in medical research?

    - Probe for
        - Trials
        - Observation studies
        - cross-sectional studies
        - social sciences studies

9. What are your views on the researcher's responsibility of planning and responding to ancillary care needs during research?

    - Probe for
        - What obligations do researchers have to participants?

- What role does context and circumstance play in determining obligation?
- How could you relate ancillary care and ideas of fairness or justice and reciprocity play out in the context of limited research funding and inadequate national health systems?

10. What guidelines does MoH have in place for biomedical researchers on the provision of ancillary care to study participants in Malawi?
11. What are your views and experiences on ancillary care provision in Malawi?
12. How could the provision of ancillary care in RCS likely create an additional burden to healthcare workers and hospitals relative to regular care?
13. What impact does the provision of ancillary care to individuals participating in biomedical research have on the health care system?
14. How does medical researchers who conduct biomedical research support with health care services within the system?
15. What do you think are the researcher's ancillary care responsibilities?

Suggestions for ancillary care in future studies

16. What are your thoughts on researchers including plans for ancillary care in the study design?
17. What would be the necessary steps to follow when deciding and considering the provision of ancillary care to study participants in Malawi and other RCS more generally?

E. <u>Research participants from selected studies</u>

Interview questions focused on the interviewee's familiarity with the concept of ancillary care and the description of the current research funding guidelines for RCS.

Experience with research participation

What research studies have you been invited to take part in?

What was your decision about participation?

Views on ancillary care

How can you describe ancillary care? or What would you describe as ancillary care in biomedical research?

Vignettes

Vignettes will be presented to participants using different scenarios to make the topic being explored clearer to participants. For each of the given vignettes, we will ask participants what they see as ancillary care and why?

It is well known that when individual volunteers decide to take part in a research study, they usually think about the benefits they might obtain from their participation, as well as the risks involved.

- Can you tell me what things you would consider important to think about before consenting to take part in a study?

On the one hand, if we think about the same considerations in terms of deciding to participate in a large research trial, such as a Covid-19 vaccine trial.

- If someone asked you if you wanted to volunteer to be part of a Covid-19 vaccine trial, what are the things that you would consider before making your decision?

If ancillary care should be provided to the study participants:

- Who should provide the care?
- What should be included as ancillary care?
- How and why should ancillary care be provided?

What are your views on the researcher's responsibility to provide ancillary care? Or what do researchers owe individual participating in biomedical research

What obligations do researchers have toward their study participants?
